# Hsp27-Actin Interaction

**DOI:** 10.1155/2011/901572

**Published:** 2011-10-10

**Authors:** Philip Graceffa

**Affiliations:** Boston Biomedical Research Institute, 64 Grove Street, Watertown, MA 02472, USA

## Abstract

Hsp27 oligomer is reported to interact with F-actin as a barbed-end-capping protein. The present study determined the binding strength and stoichiometry of the interaction using fluorescence of probes attached to Hsp27 cysteine-137. The fluorescence of acrylodan attached to Hsp27 increased 4-5-fold upon interaction with F-actin. Titration of the fluorescence with F-actin yielded a weak binding constant (*K*_*D*_^app^ = 5.3 *μ*M) with an actin/Hsp27 stoichiometry between < 1 and 6. This stoichiometry is inconsistent with an F-actin end-capping protein. Pyrene attached to Hsp27 exhibited a large excimer fluorescence, in agreement with the known proximity of the cysteine-137's in the Hsp27 oligomer. Upon interaction with F-actin the pyrene-Hsp27 excimer fluorescence was largely lost, suggesting that Hsp27 interacts with F-actin as a monomer, consistent with the acrylodan-Hsp27 results. EM images of F-actin-Hsp27 demonstrated that Hsp27 is not a strong G-actin sequester. Thus, Hsp27, *in vitro*, is a weak F-actin side-binding protein.

## 1. Introduction

Mammalian heat shock protein 27 (Hsp27) is a member of a family of small heat shock proteins that includes the eye lens protein *α*B-crystallin, the archetype of the family [1–3]. These proteins form oligomeric structures and share a conserved *α*-crystallin domain responsible for intersubunit *β*-strand-*β*-strand interactions in the basic dimer subunit. Under physiological conditions Hsp27 occurs as a polydisperse distribution of oligomers centered about a minimum 22mer [4]. Hsp27 is involved in a variety of cellular functions including molecular chaperone activity, control of apoptosis, and regulation of the actin filament cytoskeleton [1–3, 5–9].

Actin is one of the most abundant proteins, that is present in almost all eukaryotic cells. The actin filament (F-actin) is composed of actin monomers (G-actin) polymerized head-to-tail to form two intertwining helical strands, resulting in a polar filament with a “barbed end” and a “pointed end” [10]. Actin filaments are part of the cytoskeleton and their dynamic structure is involved in the motility and shape change of the cell [10]. Hsp27 has been implicated in numerous physiological and pathological processes that involve its interaction with actin and its control of actin dynamics [11–25]. However, the mechanism of the interaction between Hsp27 and actin is unclear and controversial and a binding study of the interaction between the two proteins *in vitro* has not been reported [3]. An early study of the Hsp25 (murine and avian isoform of Hsp27)—actin interaction concluded that Hsp25 was an actin filament barbed-end-capping protein based on limited evidence [26]. Since that study it has generally been assumed that Hsp25 and Hsp27 are actin barbed-end-capping proteins, although there has been little further support for this interaction model. It is also unknown if Hsp27 interacts with the actin filament as a monomer, dimer, or higher multimer.

In order to address some of these issues we have investigated the interaction between Hsp27 and actin, *in vitro*, using fluorescence probes attached to Hsp27. We concluded that Hsp27, although an oligomer in solution, binds to the actin filament, most likely, as a monomer, with an actin monomer/Hsp27 molar stoichiometry between <1 and 6. Such a stoichiometry does not support the view that Hsp27 is simply an actin filament end-capping protein. Instead, we propose that Hsp27 binds along the side of the F-actin filament.

## 2. Materials and Methods

### 2.1. Preparation of Hsp27 and Actin

Full-length Hsp27 was prepared by bacterial expression and purification using the New England Biolabs IMPACT-CN intein system, as described previously [4]. The advantage of using the intein expression system is that no extraneous N- or C-terminal residues are introduced. Additional N-terminal amino acids in expressed Hsp27 have been shown to alter the oligomeric structure of Hsp27 and other small heat shock proteins [27, 28]. The purified Hsp27 was concentrated by Millipore/Amicon Ultra centrifugal filter devices, then dialyzed and stored on ice in 2 mM Mops, 0.1 mM EDTA, 0.01% NaN_3_, 50 *μ*M PMSF, pH 7.5 (Buffer A). Hsp27 concentration was determined from the absorbance at 280 nm, after subtracting the absorbance at 320 nm, using an extinction coefficient of A_280 nm_^0.1%^ = 1.65 cm^−1^. Actin was prepared as reported [29] from rabbit skeletal muscle and stored for up to two weeks on ice as the actin monomer in G-buffer (2 mM Mops, 0.2 mM CaCl_2_, 0.2 mM ATP, 0.01% NaN_3_, pH 7.5). Actin concentration was determined from the absorbance at 290 nm minus that at 320 nm using an extinction coefficient A_290 nm_^1%^ = 6.3 cm^−1^ [30]. G-actin was polymerized to F-actin by adding NaCl and MgCl_2_ to 40 mM and 2 mM, respectively (F-buffer).

### 2.2. Fluorescence Labeling of Hsp27

The sulfhydryl reactive fluorescent probes acrylodan (6-acryloyl-2-(dimethylamino)naphthalene) (AC) and pyrene iodoacetamide (N-(1-pyrene)iodoacetamide) were purchased from Molecular Probes (Invitrogen). The single cysteine (Cys137) of Hsp27 was labeled with the above probes for several hours at 37°C as described for other probes [31]. Briefly, Hsp27 in Buffer A was reacted with a 2.5-fold molar excess of acrylodan for 3 hr or a 10-fold molar excess of pyrene-iodoacetamide for 5 hr at 37°C. The reaction was stopped with 5 mM DTT, followed by dialysis versus Buffer A to remove dissolved excess label, after centrifuging pyrene-Hsp27 to pellet the suspended, unreacted pyrene-iodoacetamide probe. The labeling ratios were determined by measuring the labeled Hsp27 concentration with the BCA protein assay [32] using unlabeled Hsp27 as a standard and measuring the attached acrylodan and pyrene concentrations from the optical absorbance with extinction coefficients *ε*_360 nm_ = 12,900 M^−1^cm^−1^[33] and *ε*_343 nm_ = 22,000 M^−1^cm^−1^ [34], respectively. The final molar labeling ratios (probe/Hsp27) were between 0.6–1.0 for pyrene-Hsp27 and 0.8–1.1 for acrylodan-hsp27.

### 2.3. Hsp27-Actin Interaction by Fluorescence Spectroscopy

Fluorescence measurements were made on a Varian Eclipse fluorometer equipped with a Varian Peltier device for temperature control. Fluorescence measurements of acrylodan-Hsp27 and pyrene-Hsp27 were made at excitation wavelengths of 395 nm and 342 nm, respectively. For fluorescence titration of acrylodan-Hsp27 with F-actin at different concentrations, separate samples were made up for each titration point and incubated overnight at 37°C before spectra were recorded. From the spectra, the fluorescence intensity was determined at 485 nm and plotted versus the total actin concentration.

The data are analyzed according to a simple binding equation, nA + B = A_n_B where A is the actin protomer in F-actin, B is Hsp27 irrespective of its oligomeric state, and n is the actin/Hsp27 molar binding stoichiometry. The binding is described by the equation: nb*ϕ* ^2^− (K_D_+ nb + a)*ϕ* + a = 0 where b is the total concentration of Hsp27 which is held constant, a is the total concentration of actin which is varied, K_D_ is the dissociation constant, and  *ϕ*  is the fractional saturation of binding sites, i.e. [AB]/nb, which is equal to the fractional increase in fluorescence enhancement and is a root of the quadratic solution to the above equation. One can extract n and K_D_ by fitting a quadratic solution to the binding equation to the data points using an unweighted, nonlinear least squares fitting procedure [35, 36].

### 2.4. Electron Microscopy of F-Actin ± Hsp27

F-actin at 150 *μ*M was diluted to 2 *μ*M in F-buffer. To some samples Hsp27 was added to 6 *μ*M and incubated for 120 min at room temperature, followed by overnight at 4°C and 120 min at room temperature. Samples were then applied to a carbon-film coated copper grid, negatively stained with 1% aqueous uranyl acetate and observed under a Philips 300 electron microscope at 60 kV.

### 2.5. Analytical Ultracentrifugation

Analytical ultracentrifugation sedimentation velocity was run and the resulting data was analyzed as described [4].

## 3. Results

The direct binding of a protein to F-actin filaments is generally determined by cosedimentation of the filaments with bound protein, followed by SDS-PAGE of the pellet and supernatant. However, Hsp27 is a large oligomeric protein which also sediments, independently of actin binding, making this technique problematic. Therefore we took an alternative approach by using Hsp27 labeled with a fluorescence probe whose signal is sensitive to the binding of actin. Two cysteine-specific fluorescence probes, acrylodan and pyrene-iodoacetamide, attached to the single cysteine (Cys137) of Hsp27 fulfilled this requirement.

### 3.1. Acrylodan-HSP27

**Figure 1:**
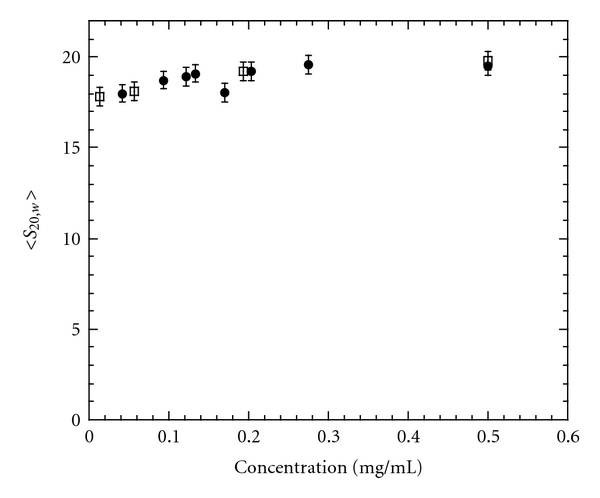
Weight average sedimentation coefficient, corrected to 20°C in water, versus concentration of Hsp27 (closed circles) or AC-Hsp27 (open squares). Buffer = 120 mM NaCl, 25 mM Tris, 2 mM MgCl_2_, 0.2 mM EGTA, 0.01% NaN_3_, 50 *μ*M PMSF, 5 mM DTT, and pH 7.5. The standard deviation of the data points is propagated from the noise in the original data set as described [37].

In order to determine the effect of the acrylodan probe on Hsp27 quaternary structure, analytical ultracentrifugation sedimentation velocity of acrylodan-Hsp27 (AC-Hsp27) was compared to that of unlabeled Hsp27 over a range of concentrations (Figure 1). No significant difference was found. Both labeled and unlabeled Hsp27 had almost identical sedimentation coefficients of close to 20 svedbergs (Figure 1), a value in agreement with our previous conclusion that Hsp27 is an oligomeric distribution centered around a minimum size of a 22-mer [4]. Thus placing a probe at Cys137 does not significantly perturb the oligomeric structure of Hsp27.

**Figure 2:**
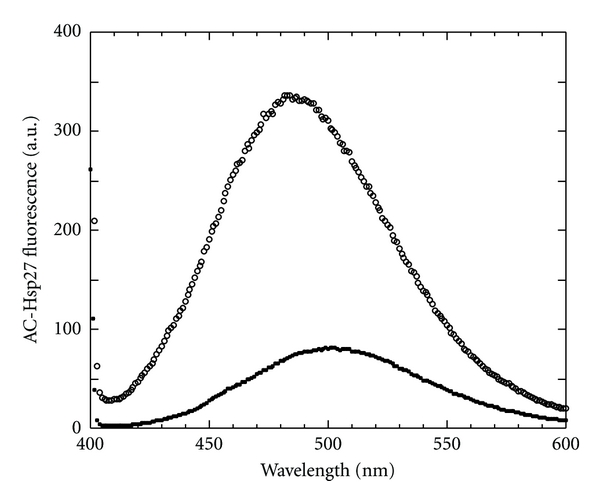
Effect of F-actin on the fluorescence spectrum of AC-Hsp27. AC-Hsp27 (1 *μ*M) at 37°C in F-buffer in the absence (closed circles) and presence (open circles) of 40 *μ*M F-actin after overnight incubation at 37°C.

Acrylodan is very sensitive to its environment [33] and, when attached to a protein, often sensitive to protein-protein interaction. As shown in Figure 2, acrylodan-Hsp27 is very sensitive to the binding to F-actin. In the presence of actin the fluorescence intensity increased about 4-5-fold and the emission spectrum was blue-shifted about 20 nm (Figure 2), changes indicative of greater probe shielding from the solvent upon interaction with actin.

**Figure 3:**
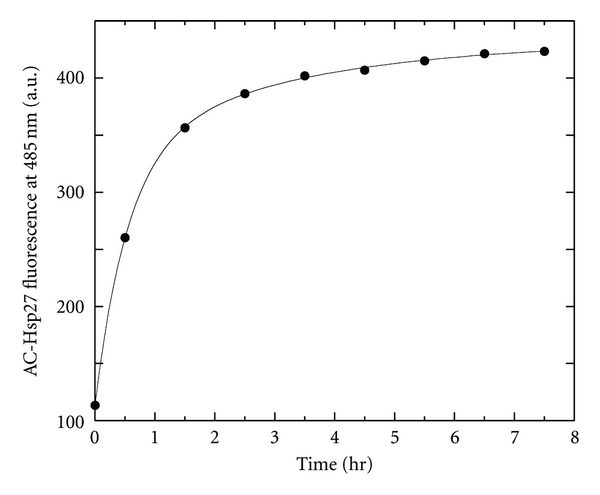
Fluorescence versus time of AC-Hsp27 plus F-actin. Fluorescence at 485 nm of 1 *μ*M AC-Hsp27 versus time after addition of 40 *μ*M F-actin in F-buffer at 37°C. The saturated fluorescence value of 432 ± 6 fluorescence units was obtained from an unweighted, nonlinear least squares double exponential fit of the data points and the root mean square deviation of the points from the fit.

In order to determine the strength and stoichiometry of the binding, the fluorescence enhancement of a fixed concentration of acrylodan-Hsp27 was titrated with increasing concentrations of F-actin at 37°C. For this determination acrylodan-Hsp27 was incubated with F-actin overnight at 37°C in order to insure complete equilibration. This was necessary since upon addition of F-actin to acrylodan-Hsp27 the acrylodan fluorescence increased slowly after an initial relatively rapid rise (Figure 3). After overnight at 37°C there was no further change in fluorescence intensity for at least 5 hours. The slow equilibration could be best fit with two exponentials (Figure 3) suggesting that at least two processes are taking place. 

**Figure 4:**
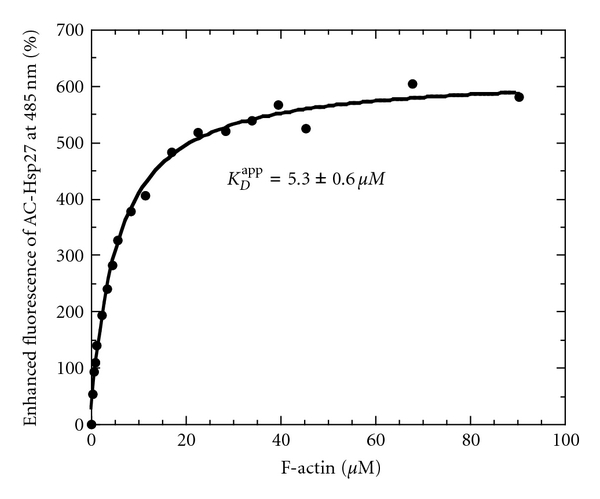
Fluorescence of AC-Hsp27 titrated with F-actin. Percent fluorescence enhancement at 485 nm as a function of actin concentration in F-buffer at 37°C. AC-Hsp27 = 1 *μ*M. The best fit of the data yielded an apparent dissociation constant *K*_*D*_^app^ = 5.3 ± 0.6 *μ*M with the actin/Hsp27 molar stoichiometry fixed at 1.0. The error in *K*_*D*_^app^ is calculated from the root mean square deviation of data points from the fit.

The titration of acrylodan-Hsp27 fluorescence intensity with F-actin was best fit with an apparent dissociation constant of *K*_*D*_^app^ = 5.3 *μ*M ± 0.6 (Figure 4), fixing the actin/Hsp27 molar stoichiometry at 1.0, indicating a rather weak interaction. Because the Hsp27-actin binding is not strong, the stoichiometry is somewhat uncertain in that the data can be fit with an actin/Hsp27 molar stoichiometry value of between <1 and 6 with a 90% confidence level using F-statistics on *χ* ^2^ values. The dissociation constant varies between 3.0 and 5.6 *μ*M over this range of stoichiometries. The fit deteriorates for stoichiometries of greater than 6 in that *χ* ^2^ values continue to increase. These results indicate that Hsp27 binds to actin all along the actin filament, each Hsp27 monomer interacting with between <1 and 6 actin subunits. Indeed, Hsp27 is an elongated molecule [4] which could well cover at least two actin subunits. A stoichiometry of less than 1 would indicate several Hsp27 molecules interacting with each actin molecule in the actin filament. This latter situation would be limited by physical crowding of Hsp27 molecules along the filament.

These results rule out that Hsp27 is binding to actin simply as a barbed-end-capping protein. The average length of an actin filament polymerized *in vitro* is about 6.7 *μ*m [38], equivalent to about 2500 actin subunits. Thus an Hsp27 22-mer [4] bound at the barbed end of each filament would result in an actin/Hsp27 molar stoichiometry of greater than 100. For smaller oligomers the stoichiometry would be higher, all values much larger than we have estimated from the fluorescence titration in Figure 4.

### 3.2. Pyrene-HSP27

Although we have ruled out that Hsp27 binds to actin simply as an end-capping protein from the acrylodan-Hsp27 results, the results do not allow us to know the size of the Hsp27 that is bound to actin. That is, does it bind to actin as a monomer, a dimer, or a larger multimer? We have used the fluorescence of pyrene-Hsp27 to aid us in this determination. The pyrene probe can be a proximity probe, in that when two pyrene moieties are in close proximity they may stack in the excited state which results in an excited state dimer, called an excimer. Pyrene excimer fluorescence is a broad band centered around 500 nm, quite different from the narrower doublet band of the pyrene monomer moiety centered at about 400 nm [39, 40].

**Figure 5:**
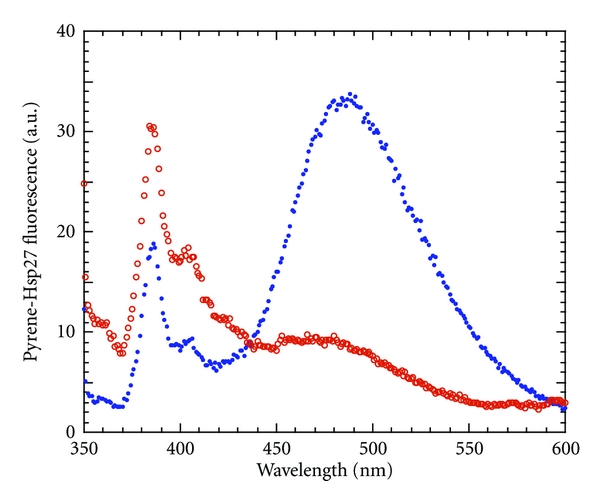
Fluorescence spectrum of pyrene-Hsp27 upon interaction with F-actin. Pyrene-Hsp27 (1 *μ*M) in the absence (blue closed circles) and presence (red open circles) of 5-fold molar excess of F-actin at 37°C in F-buffer.

Cys137 of Hsp27 (Cys141 of Hsp25) has been shown to be close to Cys137 of a neighboring Hsp27 molecule in the basic dimeric building block of the Hsp27 oligomer by spin-spin ESR spectroscopy [41, 42] and by disulfide cross-linking [26, 42–44]. We thought that we might be able to exploit pyrene excimer fluorescence to monitor this interaction. In agreement with this proximity the fluorescence spectrum of pyrene-Hsp27 showed a relatively intense excimer band compared to that of the band of the pyrene monomer (Figure 5). Upon interaction with F-actin the intensity of the excimer fluorescence of pyrene-Hsp27 dropped dramatically with a smaller concomitant increase in that of the pyrene monomer (Figure 5). The remaining excimer fluorescence in the presence of actin is most likely due to pyrene-Hsp27 that is not bound to actin since the 5-fold molar excess of actin used in this experiment is not sufficient to complex all of the Hsp27 (Figure 4). The concentration of F-actin used was limited by its Rayleigh light scattering which overlaps with the pyrene monomer band. Therefore it is clear that, upon binding of pyrene-Hsp27 to F-actin, the excimer fluorescence is almost entirely lost and this loss is most likely due to a dissociation of pyrene-Hsp27 into monomer molecules attached to actin and thus no longer able to form excimers. These results, together with those of AC-Hsp27, are consistent with Hsp27 binding to actin as a monomer with an actin/Hsp27 molar stoichiometry between <1 and 6.

However we cannot entirely rule out that the loss in pyrene-Hsp27 excimer fluorescence upon actin binding is due to a conformational change around Cys137 such that pyrene moieties are no longer able to properly stack for excimer formation. In that case the results would also be consistent with a pyrene-Hsp27 binding to F-actin as a dimer or a multimer with the loss of excimer due to a long-range allosteric conformational change in Hsp27 resulting in the loss of excimer.

### 3.3. Electron Microscopy of the Actin-HSP27 Complex

**Figure 6:**
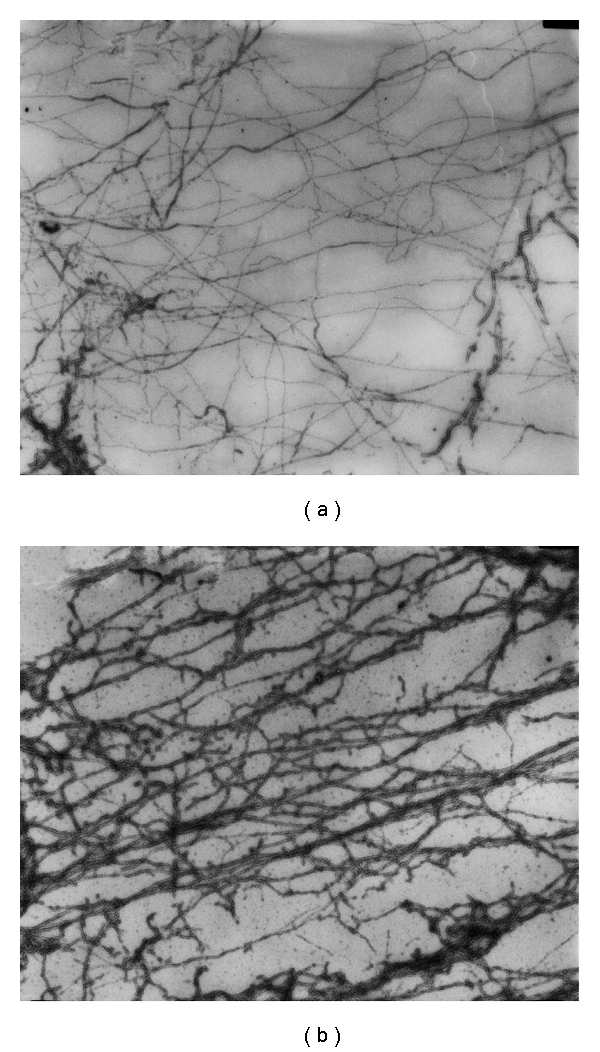
Electron microscopy of F-actin ± Hsp27. Negative staining images of 2 *μ*M actin (a) and 2 *μ*M actin plus 6 *μ*M Hsp27 (b) incubated overnight in F-buffer. Magnification equals x 15960. Images courtesy of Dr. Katsuhide Mabuchi.

It has been concluded from indirect evidence that Hsp27 is a strong actin monomer sequestering protein [24]. It was calculated that 3-4 actin monomers bound tightly to each Hsp27 monomer within the Hsp27 oligomer, with a dissociation constant of 20 nM [24] at 25°C. In order to rule out such an interaction accounting for our findings we observed electron microscopic images of F-actin filaments in the absence and presence of a threefold molar excess of Hsp27 under the F-actin ionic conditions used above. Strong Hsp27 sequestration of actin monomers in equilibrium with actin filaments would result in a dissolution of the filaments upon incubation of the filaments with Hsp27. Our electron microscopic images (Figure 6) showed that, although Hsp27 results in some aggregation of F-actin filaments, there is no significant dissolution of the filaments, indicating no significant actin monomer sequestration by Hsp27. Thus such a mechanism cannot account for our fluorescence observations above.

## 4. Discussion

This study investigated the equilibrium binding parameters of the *in vitro* interaction between Hsp27 and F-actin and concluded that, *in vitro*, Hsp27 is not a simple actin barbed-end-capping protein, as is generally assumed. Rather, it is concluded that Hsp27 binds weakly along the side of the actin filament as a monomer with an actin/Hsp27 molar ratio between <1 and 6. A previous work also concluded that monomeric but not oligomeric Hsp25 interacted with actin [45]. However, we cannot entirely rule out that Hsp27 is a barbed-end-capping protein in addition to being an actin side-binding protein, which has been shown to be the case for another actin-binding protein, vinculin [46].

The fitting of the binding data assumes a rather simple binding model of Hsp27 monomer in solution in equilibrium with actin-bound Hsp27. However, Hsp27 is primarily an oligomer in solution [4]; therefore the apparent dissociation constant from the fit reflects the difference in binding between Hsp27 monomer bound to the oligomer compared to its binding to the actin filament. Thus we can only conclude that Hsp27 binds weakly to actin compared to its binding to itself in the Hsp27 oligomer. The strong binding of Hsp27 to itself might reflect a slow rate of dissociation of the monomer (or dimer) from the Hsp27 oligomer and account, at least in part, for the slow attainment of equilibrium of its binding to actin. Alternatively, the first step in the binding sequence might be the attachment of the Hsp27 oligomer to the actin filament which then slowly “peels off” Hsp27 monomers. 

Another work has also concluded that Hsp27 is not a barbed-end-capping protein by the effect of Hsp27 on actin polymerization properties [24]. That work concluded that Hsp27 is primarily a strong actin monomer sequestering protein, tightly binding 3-4 actin monomers per Hsp27 molecule in the Hsp27 oligomer [24]. However, our electron microscopic images of F-actin plus Hsp27 demonstrate that Hsp27 does not dissolve actin filaments and thus is not a strong actin monomer sequestering protein. Another recent work has also concluded, from actin polymerization studies, that Hsp27 is inefficient in sequestering actin monomers in the presence of actin filaments [47]. That work [47] and the present work employ Hsp27 without any extra residues at either end of the molecule whereas the previous work [24] used Hsp27 with extra residues at the N-terminus. It has been shown that extra N-terminal residues can modify the oligomeric structure of Hsp27 and other similar small heat shock proteins and thus possibly modify its function [27, 28]. This might be the source of the above discrepancies.

In conclusion, *in vitro*, Hsp27 is not a simple barbed-end-capping protein but instead primarily binds to the sides of the actin filament. Compared to its binding to itself in the Hsp27 oligomer, Hsp27 binds weakly to actin. The functional consequence of such a weak and slow-to-reach-equilibrium interaction awaits further investigation.

## Abbreviations

Hsp27: Heat shock protein 27
DTT: Dithiothreitol
Mops: 3-(N-morpholino)propanesulfonic acid
PMSF: Phenylmethylsulfonyl fluoride
Acrylodan: 6-acryloyl-2-(dimethylamino)naphthalene
Acrylodan-Hsp27 or AC-Hsp27: Hsp27 labeled with acrylodan
Pyrene Iodoacetamide: (N-(1-pyrene)iodoacetamide)
Pyrene-Hsp27: Hsp27 labeled with pyrene iodoacetamide
a.u.: Arbitrary units.
